# Changes in the Characteristics of Kidney Cancer Detection During the COVID-19 Pandemic

**DOI:** 10.3390/cancers17132150

**Published:** 2025-06-26

**Authors:** László Rumi, Árpád Szántó, Dániel Bányai, Éva Szabó, Antal Zemplényi, Szabolcs Bellyei, Emese Mátyus, Dóra Hubai, János Girán, István Kiss, Éva Pozsgai, Árpád Boronkai

**Affiliations:** 1 Urology Clinic, Clinical Center, University of Pécs, Munkácsy Mihaly Street 2, 7621 Pécs, Hungary; 2 Department of Otorhinolaryngology, University of Pécs Clinical Center, Munkácsy Mihaly Street 2, 7621 Pécs, Hungary; 3 Department of Otorhinolaryngology Head and Neck Surgery, Szent György University Teaching Hospital, Fejér County, Seregélyesi Street 3, 8000 Székesfehérvár, Hungary; 4 Center for Health Technology Assessment and Pharmacoeconomics Research, Faculty of Pharmacy, University of Pécs, Rókus Street 2, 7624 Pécs, Hungary; 5 Department of Oncotherapy, University of Pécs Clinical Center, Édesanyák Street 17, 7624 Pécs, Hungary; 6 Department of Public Health Medicine, University of Pécs Medical School, Szigeti Street 12, 7624 Pécs, Hungary; 7 Department of Primary Health Care, University of Pécs Medical School, Rákóczi Street 2, 7623 Pécs, Hungary

**Keywords:** kidney cancer, COVID-19, detection, discovery, incidental, non-incidental, female, advanced-stage, predictive factor, renal cancer

## Abstract

**Background/objectives**: The COVID-19 pandemic affected cancer care globally. Our objective was to analyze the demographic and clinical characteristics of kidney cancer (KC) patients between the pre-COVID-19 and COVID-19 periods. We also aimed to assess how KC was discovered—incidentally or symptomatically—and identify factors predicting the mode of discovery and advanced-stage disease. **Methods**: This retrospective study analyzed data from 400 patients aged 18 years or older diagnosed with kidney cancer (KC) at a large regional Hungarian clinical center during two time periods: the pre-COVID-19 period (1 January 2019 to 15 March 2020) and the COVID-19 period (16 March 2020 to 13 May 2021). Demographic and clinical information, including the mode of cancer discovery, was collected for all patients. **Results**: During the pandemic, monthly kidney cancer diagnoses declined by 10.3%. The proportion of female patients rose significantly from 31.9% to 42.9% (*p* = 0.023). Incidental tumor detection decreased from 82.4% to 72.4% (*p* = 0.018), while symptomatic presentation increased from 14.2% to 19.4%, although not significantly (*p* = 0.166). Non-incidental detection was associated with a 3.42-fold increase in odds of advanced cancer pre-pandemic and a 2.03-fold increase during the pandemic. Symptomatic presentation raised these odds by 4.51 and 2.76 times, respectively. **Conclusions**: Our study revealed changes in kidney cancer detection during the pandemic, including a rise in the proportion of female patients and a decline in case numbers, likely due to reduced incidental findings. Non-incidental discovery and symptom presence remained predictors of advanced-stage disease, although the odds were lower. Various factors—such as changes in healthcare access and gender-related differences in health-seeking behavior—may possibly explain these changes. Our findings support the critical role of incidental detection in high-risk populations.

## Simple Summary

The COVID-19 pandemic disrupted healthcare systems globally. This study aimed to assess how these changes affected kidney cancer detection by comparing the data of patients before and during the pandemic at a large Hungarian clinical center. Medical records from 400 patients were analyzed to understand how the cancer was discovered—either incidentally (by chance during unrelated tests) or non-incidentally (for example, after symptoms appeared)—and what factors were linked to advanced-stage disease. During the pandemic, monthly kidney cancer cases dropped by 10.3%, and the proportion of female patients increased from 31.9% to 42.9%. Incidental detections fell from 82.4% to 72.4%, while non-incidental cases increased slightly. Non-incidental detection was associated with a two-to-threefold higher risk of advanced-stage cancer. Our findings suggest the pandemic uniquely impacted kidney cancer cases, with reduced incidental detection likely leading to later diagnoses. The rise in female cases may reflect gender differences in health-seeking behavior during the pandemic.

## 1. Introduction

Kidney cancer (KC) is the third most common urological cancer globally, resulting in approximately 180,000 deaths each year worldwide [1]. Cancer incidence and mortality rates surpass the EU averages for both genders in Hungary, with KC being the 7th most common cancer overall [2]. Renal cell carcinoma (RCC) represents the predominant solid tumor found in the kidney, constituting about 90% of all cancers in the kidney.

The incidence of KC is increasing, showing significant disparities between genders, with KC being the ninth most common cancer in men and the 14th in women [3]. Epidemiological data indicate that men are nearly twice as likely to develop RCC, and they often present with more aggressive disease than women [4,5]. These differences are likely influenced by both biological factors (e.g., hormonal differences) and behavioral elements, including healthcare-seeking patterns and lifestyle factors such as smoking [4,5]. The growing number of cases may be partly attributed to the increased incidental discovery of renal masses during abdominal imaging conducted for vague musculoskeletal or gastrointestinal issues [1]. Despite advancements in the detection and treatment of KC, there is a paradoxical rise in mortality rates in certain European nations [6].

Kidney cancer frequently presents as an incidental finding of a localized mass during imaging, with some studies indicating that up to 85% of cases are detected incidentally [7]. A large multicenter study in the UK found that 87% of patients with stage Ia and 36% with stage III or IV disease were diagnosed incidentally [8]. Only 30% of RCC cases are identified through symptoms, as small masses typically do not elicit any [9]. Given that symptomatic presentations are often linked to more advanced tumor stages, it is not surprising that patients with incidental RCC diagnoses experience better outcomes compared to those diagnosed through other means, as highlighted in a study from the UK [8].

The COVID-19 pandemic significantly disrupted healthcare delivery worldwide, particularly impacting cancer care and patient pathways [10,11]. A number of studies, including a systematic review, identified key issues, like reductions in cancer treatments and surgeries, delays in radiotherapy, and the cancellation or rescheduling of outpatient visits during the pandemic [12]. These findings emphasize that prolonged delays in cancer treatment increase the risk of mortality, highlighting the vital need for timely intervention [10]. In Hungary, as in many European countries, the provision of healthcare services was affected during the pandemic due to lockdowns and a focus on care provision for COVID-19-infected patients [13].

Changes in renal cancer diagnoses and stage migration have been reported internationally. A decline in the number of RCC cases was observed during the pandemic, especially for small tumors (T1a/T1b), likely due to their incidental detection during routine imaging [14]. A study in the UK found no increase in clinical or pathological upstaging among patients who had deferred surgery compared to pre-COVID practices; however, referrals to the specialist multidisciplinary team meetings dropped significantly by 53% during the pandemic [15]. Similarly, a study in the Netherlands reported a 15% decrease in new RCC diagnoses during the first wave of COVID-19 (weeks 9–22, 2020) due to diagnostic delays [16]. In Canada, Janes et al. reported a significant decline in new RCC diagnoses during the pandemic, with a notable shift toward more advanced-stage presentations at the time of detection [17]. A recent German tertiary center reported increased mortality and higher pathological stages in renal cancer patients undergoing nephrectomy during the pandemic period [18].

Given these findings [16,17,18], it is plausible to hypothesize that the pandemic altered RCC detection modes and patient profiles. While it is logical to assume that incidental cancer detection declined due to the reduction in imaging and consultations, few studies have empirically evaluated these changes across the diagnostic pathway, particularly in Central-Eastern Europe, where healthcare system differences may yield distinct effects.

Therefore, it was our objective to analyze the demographic and clinical characteristics of kidney cancer patients, comparing data between the pre-COVID-19 and COVID-19 pandemic periods at a large Hungarian tertiary care center. We also aimed to evaluate how the cancer was discovered—whether incidentally or with symptoms characteristic of cancer—and to identify predictive factors associated with the mode of discovery and advanced-stage cancer.

## 2. Methods

### 2.1. Setting

The research was conducted at the University of Pécs Clinical Center, specifically within the Urology Clinic (UP UC) in Pécs, Hungary. Serving as a regional facility, the UP UC not only provides cancer care for patients in Baranya County but also cares for individuals from adjacent counties throughout the Transdanubian region. Nearly 4000 surgical interventions are performed annually at the UP UC, and pioneering work in nephron-sparing surgeries for kidney cancer patients has been carried out over the past decades. The University of Pécs Clinical Center houses a specialized cancer center comprising an inpatient unit, a day oncology unit, and a radiotherapy unit for the provision of complex care of cancer patients.

Prior to the research procedure, our study obtained ethical approval from the Regional Ethical Committee (Reference number: 9389–PTE 2022).

### 2.2. Impact of COVID-19 Pandemic Regulations in Hungary

Hungary reported its first symptomatic COVID-19 cases in early March 2020, prompting a national safety crisis declaration on March 11. Shortly after, schools closed, elective procedures were postponed, and dental care was limited to emergencies. Although elective surgeries resumed under specific conditions from May to November 2020, the country remained in a state of preparedness. Oncological, emergency, cardiological, and reproductive procedures continued without interruption. Although scheduled outpatient visits were curtailed during the pandemic, diagnostic pathways—including imaging—remained active for acute or ongoing health issues when delays could have jeopardized patient safety, thereby even allowing for incidental findings of tumors to occur. All restrictions were lifted on 13 May 2021, marking a return to pre-pandemic healthcare guidelines.

### 2.3. Study Design

This study was an observational, retrospective analysis. The inclusion criteria encompassed all patients aged 18 and older who attended the UP UC from 1 January 2019 to 13 May 2021, and had a histological confirmation of kidney cancer. Patients were included based on diagnostic criteria if they received an International Classification of Diseases, 10th Revision (ICD−10) code of C64H0. Exclusion criteria were set to eliminate individuals with any other tumors diagnosed within five years prior to the kidney cancer diagnosis, as well as those with secondary tumors. All collected data pertain specifically to renal cell carcinoma (RCC), identified using the ICD-10 code C64H0. Therefore, the terms “kidney cancer” and “RCC” are used interchangeably throughout the manuscript. Urothelial and other tumors in the kidney were excluded.

The full study period was separated into two distinct intervals, categorized by their occurrence before or during the COVID-19 pandemic. The first interval, from 1 January 2019 to 15 March 2020, was labeled the pre-COVID-19 period, while the second interval, from 16 March 2020 to 13 May 2021, was identified as the COVID-19 period. The designation of these periods was informed by regulations issued by the Hungarian National Directorate General for Hospitals, which outlined the changes in healthcare delivery during the pandemic, as mentioned above.

Using automated data collection methods, the electronic database of the University of Pécs Clinical Center was screened for all patients who fulfilled the inclusion criteria, resulting in a total of 400 patients aged 18 and older diagnosed with kidney cancer throughout the entire study period. Demographic information was gathered, including sex distribution, age at the time of the visit to UP UC, and place of residence. Following this, manual data collection was undertaken to obtain additional information, such as the distance of patients’ residences from the clinic, types of comorbidities, tobacco usage, types and number of primary symptoms at presentation, histological cancer type and stage, the specialty of the physician at first contact, and mode of cancer detection. Since tobacco use is common among RCC patients, it was recorded as an established principal RCC risk factor and potential modifier of tumor presentation and clinical characteristics [19]. Symptom presence and type were extracted from physician notes recorded during the initial consultation, accessed through the clinic’s electronic medical records system. All symptoms reported by patients were documented. The chief complaints, diagnoses, and comorbidities were categorized according to the ICD−10 classification system. The distance between the patient’s place of residence and the UP UC was calculated using Google Maps, where the shortest route in kilometers was determined. Comorbidities were registered based on the list of diseases of the Charlson Comorbidity Index (CCI), which predicts 10-year survival in patients with multiple comorbidities [20], and were subsequently categorized into two groups: patients with 0−4 points and patients with at least 5 points or more. During the study periods, both open and laparoscopic surgeries were performed. Robot-assisted surgery was not available in our region at the time.

Regarding the mode of discovery of the KC, incidental/non-incidental and asymptomatic/symptomatic cases were recorded.

Incidental cases were defined as those in which imaging was performed for reasons unrelated to cancer. These included patients receiving urgent or emergency care (e.g., for acute gastrointestinal symptoms, urinary retention) or undergoing routine management of chronic conditions such as hypertension, diabetes, or cardiovascular disease.

Non-incidental cases included all patients presenting with symptoms suspected of kidney cancer (e.g., hematuria, flank pain), as well as those under urological surveillance for potential tumors, such as individuals with kidney cysts, which can occasionally undergo malignant transformation and therefore require regular monitoring.

**Figure 1:**
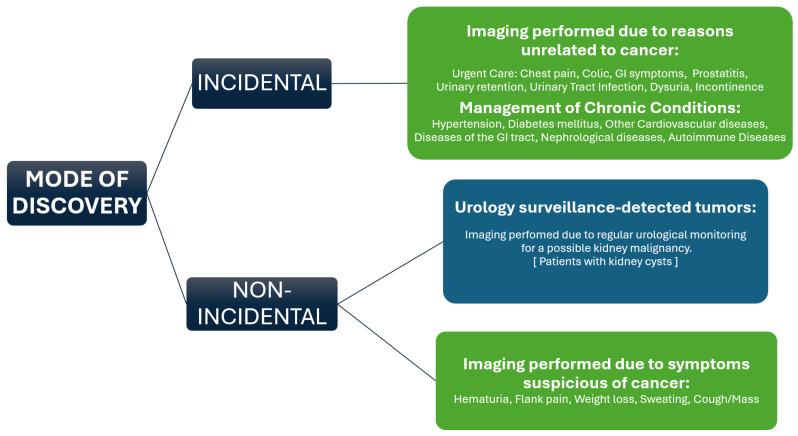
Categories and modes of discovery of kidney cancer.

The specific categories of kidney cancer detection modes are detailed in Figure 1.

The main objectives of this study were to examine how kidney cancer was detected and to compare demographic and clinical characteristics before and during the COVID-19 pandemic. Secondary objectives included identifying potential predictors of incidental diagnoses and advanced-stage cancers across the two timeframes. The analysis of predictive factors was based on the variables listed in Table 1.

### 2.4. Data Analysis

To answer the study’s research questions, a data analysis framework was designed incorporating both descriptive and exploratory methods. Demographic and clinical features of kidney cancer cases were summarized using frequency tables. The Chi-square test was used to assess statistical significance, with a *p*-value of <0.05 considered statistically significant. Following the assessment of normality using the Shapiro–Wilk test, an independent-samples Mann–Whitney *U* test was performed to evaluate differences in the distribution of monthly KC diagnoses between the two study periods. Binary logistic regression analysis was conducted to identify factors that predict contacting a physician in the advanced stages (III–IV) of the disease. A Z-test was applied to assess differences in odds ratios across the two study periods. Statistical analyses were performed using the statistical software Jamovi 2.6.26.

## 3. Results

### 3.1. Distribution and Baseline Characteristics of Patients with Kidney Cancer Before and During the COVID-19 Pandemic

**Figure 2:**
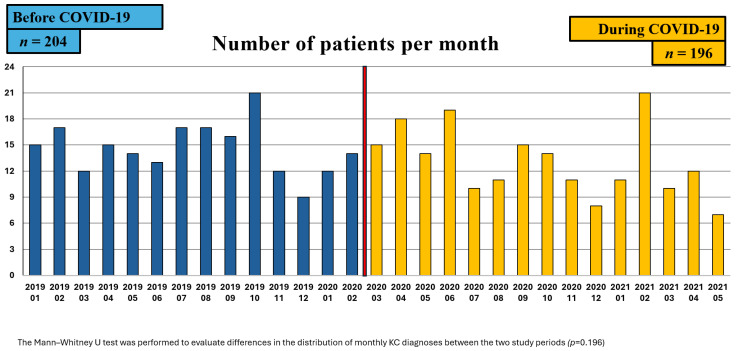
Monthly and total numbers of RCC patients before (*n* = 204) and during (*n* = 196) the COVID-19 pandemic.

A total of 204 patients were diagnosed with kidney cancer before the pandemic (1 January 2019 to 15 March 2020), and 196 patients during the pandemic (16 March 2020 to 13 May 2021). During the pandemic period, a 10.3% decrease in the average monthly number of kidney cancer diagnoses was observed. Both periods showed variability in monthly case numbers, without a consistent trend (Figure 2). The highest number of cases prior to the pandemic occurred in October 2019, while during the pandemic, the peak was observed in February 2021.

The proportion of female patients increased from 31.9% to 42.9%, while the percentage of male patients decreased from 68.1% to 57.1%, a statistically significant change (*p* = 0.023) during the pandemic. Additionally, a significantly larger proportion of patients resided within a 40 km distance from the clinic during the pandemic compared to the pre-pandemic period (41.2% vs. 51%; *p* = 0.048). There was also a notable increase in the proportion of patients with fewer comorbidities during the pandemic (42.2% vs. 52% pre-pandemic and during the pandemic; *p* = 0.048) (Table 1).

In contrast, no significant differences were observed between the two study periods in terms of age distribution, type of residence, smoking characteristics, specialty of the initially consulted physician, initial imaging test distribution and the distribution of early versus advanced-stage cancer, although a slight increase in advanced cancer cases (from 21.6% to 25%) was noted (Table 1).

### 3.2. Modes of Tumor Detection and Influencing Factors Before and During the COVID-19 Pandemic

Symptom type and the detecting physician’s specialty are clinically important as they affect the speed and accuracy of kidney cancer diagnosis. Symptoms like hematuria often trigger urgent referrals to urologists and timely imaging, leading to earlier detection, while nonspecific symptoms such as flank pain may cause varied referrals and potential delays. Consequently, symptom type influences diagnostic pathways, stage at diagnosis, and outcomes. Conversely, incidental findings by non-urologists may lead to delays in diagnosis or varied diagnostic pathways, impacting treatment decisions and prognosis.

**Figure 3:**
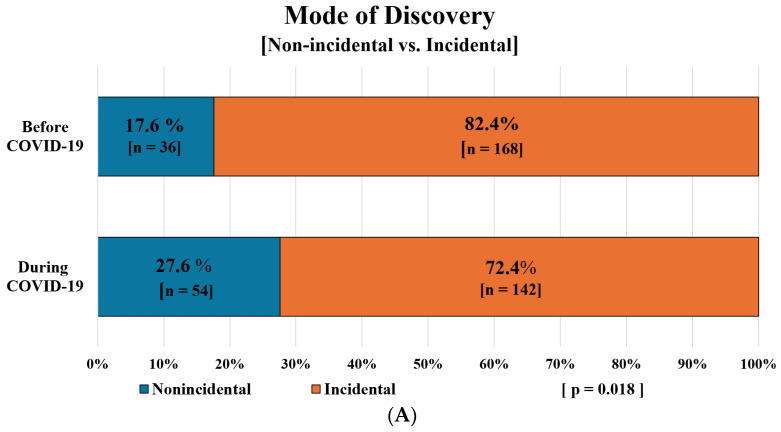

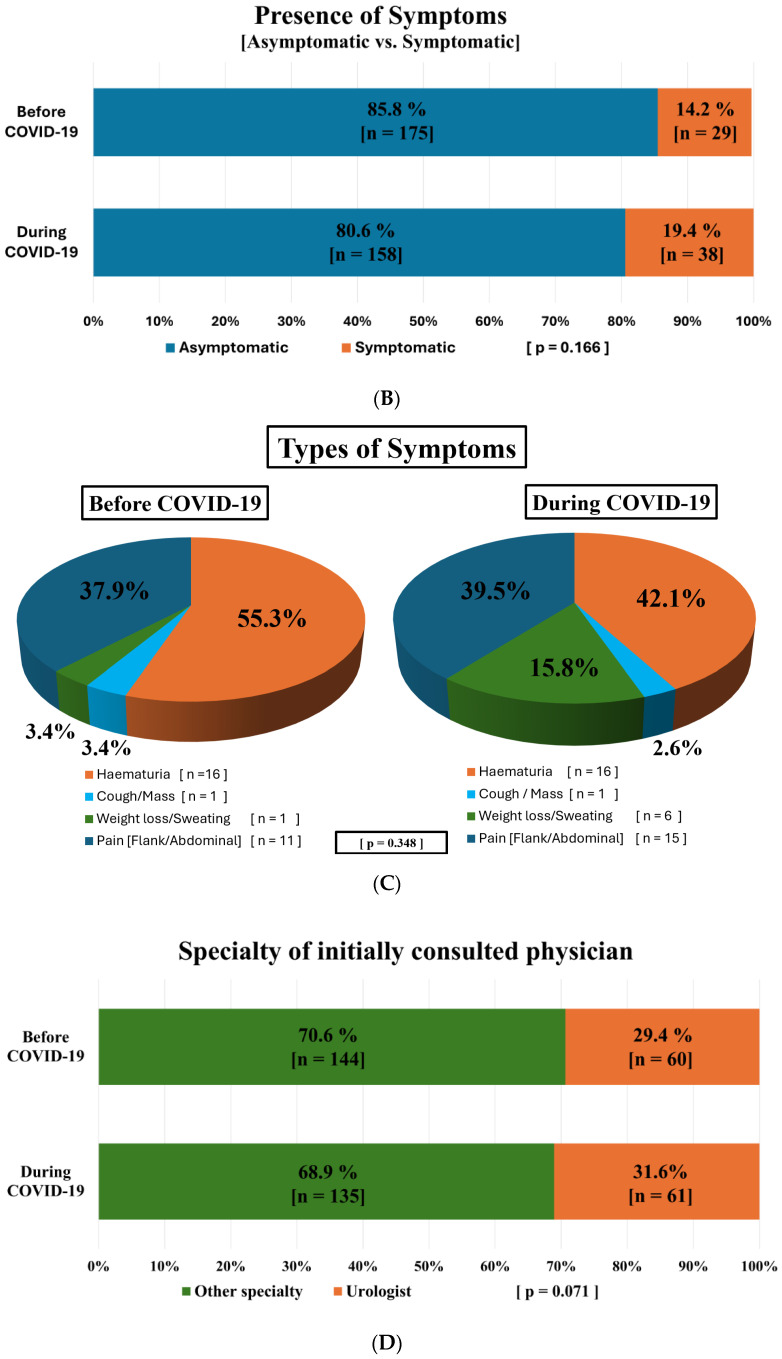
The distribution of (**A**) mode of discovery, (**B**) presence of symptoms, (**C**) types of symptoms of KC, and (**D**) specialty of initially consulted physician before and during the pandemic.

In terms of tumor detection, we found that the majority of the patients’ tumors were detected incidentally during both study periods, while patients presenting with symptoms represented a small minority across both periods (Figure 3A,B). Among those with symptoms, hematuria was the most common, reported by 55.3% of symptomatic patients before the pandemic and 42.1% during the pandemic, followed by flank/abdominal pain at 37.9% and 39.5%, respectively (Figure 3C).

The proportion of tumors discovered incidentally decreased significantly during the pandemic, dropping from 82.4% to 72.4% (*p* = 0.018). Conversely, the percentage of patients presenting with symptoms saw a slight increase, from 14.2% in the pre-COVID period to 19.4% during the pandemic; however, this change was not statistically significant (*p* = 0.166). (Figure 3A,B). No significant differences were observed in the types of symptoms reported between the two study periods or the specialty of the initially consulted physician (urologist or other) between the two study periods (Figure 3C,D).

To further investigate the patterns of characteristics among kidney cancer patients, we analyzed demographic and clinical parameters that may influence incidental discoveries between the two study periods. The pandemic period resulted in significant differences in cancer stage and the specialty of the initially contacted physician among patients with incidental findings of kidney cancer, as detailed in Table 2.

During the pandemic, there was a notable increase in the proportion of advanced-stage cancer among patients whose tumors were discovered incidentally, rising from 17.3% in the pre-pandemic period to 21.1% during the pandemic (*p* = 0.000). A small but significant decline was also observed in the percentage of patients initially contacting a urologist among incidental cases, dropping from 25.6% pre-pandemic to 25.4% during the pandemic (*p* = 0.000; Table 2).

No statistically significant relationship was found between the distribution of the age groups, residence type, tobacco use, and number of comorbidities/CCI points among patients with incidental findings between the two study periods. (Table S1).

### 3.3. Predictive Factors for Advanced Tumor Stage and Incidental Discovery Before and During the COVID-19 Pandemic

We examined whether predictive factors for incidental discovery and advanced-stage (stages III–IV) cancer could be identified among the parameters analyzed in the study, as well as any differences between the two study periods.

Logistic regression analyses revealed that non-incidental discovery and the presence of symptoms independently predicted higher odds of seeking medical attention at an advanced stage of the disease. Additionally, consulting a physician other than a urologist significantly increased the odds of incidental discovery.

Consulting a physician other than a urologist significantly increased the odds of incidental discovery of kidney tumors, with similar effects observed during both study periods (2.6 times in the pre-COVID period and 2.54 times during the pandemic).

A non-incidental discovery of the tumor raised the odds of having advanced cancer by 3.42 times before the pandemic and 2.03 times during it. Similarly, the presence of symptoms increased the odds of advanced-stage cancer by 4.51 times pre-pandemic and 2.76 times during the pandemic.

Therefore, patients with non-incidental discoveries of kidney cancer were less likely to contact a physician at an advanced stage of the disease during the pandemic compared to the pre-COVID period (Table 3). Additionally, patients presenting with symptoms also demonstrated a decreased likelihood of consulting a physician at an advanced stage during the pandemic compared to the pre-pandemic era (Table 3).

However, it should be noted that in both cases (Table 3A,B), the differences between the odds ratios were not statistically significant and should be interpreted with caution.

## 4. Discussion

Our study revealed notable associations with changes in the impact of the COVID-19 pandemic on the detection patterns and characteristics of kidney cancer patients. We found a significant change in the gender distribution and number of comorbidities during the pandemic. The overall decline in kidney cancer cases was associated with a decrease in incidental detections. We also identified non-incidental discovery and symptom presence as predictive factors for advanced-stage KC; however, their predictive strength appeared weaker during the pandemic, although this difference was not statistically significant.

Previous studies have indicated that the management of major urological cancers was significantly impacted during the COVID-19 pandemic, with most European centers reporting these effects [21,22]. Uro-oncological consultations for newly diagnosed cancers fell by 55−71% [22], and uro-oncological procedures were delayed in 50% of cases, which coincided with a 66% drop in new cancer diagnoses [23]. A UK study conducted between 23 March and 10 May 2020, reported a 47% reduction in specialist multidisciplinary meetings for newly diagnosed kidney cancer patients [15]. In contrast, a study from the Netherlands noted only a 15% decrease in newly diagnosed renal cell carcinoma cases during the first COVID-19 wave [16], likely due to the longer timeframe examined. Similarly, our research found an approximately 10% decline in newly diagnosed cases. Complementing the findings from Western Europe—however, not solely focused on kidney cancer cases—a multicenter cross-sectional study from Croatia reported that 25% of cancer patients undergoing systemic therapy experienced pandemic-related impacts on their cancer care, with 10.2% experiencing treatment changes or discontinuation due to COVID-19 restrictions [24]. This regional data aligns with our observations, highlighting that the pandemic’s indirect effects on oncology care were widespread across Europe.

The risk of developing kidney cancer is approximately twice as high in men as in women, a trend observed consistently across various regions and age groups over time [25,26]. In our study, we also noted a male predominance in KC during both study periods; however, there was a significant rise of about 10% in the proportion of female patients during the COVID-19 pandemic, alongside a decrease in male patients. Gender differences have primarily been attributed to biological factors, such as the activation of the estrogen receptor beta, which may act as a tumor suppressor, and to a lesser extent, lifestyle factors including smoking and higher than normal BMI [27,28]. However, since risk factors typically require a longer time to affect KC development, our observation of a notably higher female ratio during the pandemic suggests an association with other underlying mechanisms may be at play beyond these risk factors: possibly the increased health consciousness of women compared to men and the effect of the pandemic on health consciousness in general. Beyond heightened health consciousness, other factors may have contributed to the increased proportion of female patients during the pandemic. Gender differences in referral patterns—whether due to more frequent acute care use by women or differential screening access—could also play a role [29]. Women may have been more proactive in seeking care despite lockdowns or may have been prioritized differently in diagnostic pathways [30]. Additionally, behavioral changes such as reduced mobility and increased telehealth engagement among women have been documented, possibly affecting presentation timing and referral patterns [31]. These hypotheses underscore the complexity of gender dynamics during crisis periods and indicate that further focused studies are needed to clarify the mechanisms behind the shift.

According to studies, although individual health consciousness varies widely based on factors such as age, education, culture, and personal experiences, research has shown that women were more health-aware, more likely to seek regular medical check-ups, engage in preventive health behaviors, and prioritize health and wellness compared to men [32,33]. Previous publications have also highlighted that awareness regarding cancer played a crucial role in promoting behaviors that facilitate early detection [34], and public awareness of health conditions was reported to increase during the pandemic via extensive media coverage, if mostly only focused on the COVID-19 infection [35]. A recent study using large-scale data revealed a notable surge in searches for COVID-19 information during this period [35]. Thus, it is plausible that the heightened awareness of COVID-19 infection among certain individuals, particularly women, may have unintentionally led them to become more mindful of their overall health, which could be associated with an increased tendency to present to a doctor with other symptoms as well.

The pandemic intensified existing social and geographical health disparities, where vulnerable populations—such as the elderly, people with chronic health conditions, and those from lower socioeconomic backgrounds—faced greater obstacles in accessing healthcare [36]. These challenges were particularly evident among people with lower educational attainment and those who were single or widowed [13,36,37]. Therefore, these factors may help explain our findings, which indicated a notable decrease in the number of patients with multiple comorbidities (over 4 points on the CCI) diagnosed with kidney cancer during the pandemic, as well as an increase in patients living closer to the clinic. Individuals with chronic health issues residing further from healthcare centers, likely in rural areas, have been reported to experience more difficulties when accessing healthcare services during the pandemic [36]. Additionally, concerns about contracting COVID-19 may also have led some individuals with multiple comorbidities to avoid seeking care in healthcare facilities [38]. While many countries implemented telemedicine to facilitate contact with healthcare professionals during lockdowns and restrictions [39], its role in imaging-based detection of kidney cancer was inherently limited. However, telemedicine may have supported continuity of care and accelerated initial consultations for symptomatic patients, potentially leading to earlier imaging and diagnosis in some cases [40].

Countries with higher median incomes often report a greater incidence of kidney cancer, likely due to more frequent use of abdominal imaging, which results in increased incidental findings of asymptomatic small renal masses [7,41,42,43]. Fewer than 15% of KC cases are diagnosed based on classic symptoms such as hematuria, flank pain, and abdominal masses [8,9]. In fact, classic symptoms are only found in 4−17% of KC cases, while indicators like weight loss, anemia-related fatigue, and cough are more commonly associated with advanced metastatic cancer [8,9]. In our study, we found that around 16.7% of the patients presented with classic symptoms of KC; most of the patients presented with hematuria and flank/abdominal pain and there was no significant difference between the proportion of patients with KC symptoms before and during the pandemic, although a slight increase in the proportion of symptomatic patients could be observed.

A prospective study conducted in the UK examined the occurrence and characteristics of both symptomatic and incidental diagnoses of renal cell carcinoma [8]. The findings revealed that a majority of patients (60%) were diagnosed incidentally [8], with 87% of those presenting with stage Ia and 36% with stage III or IV disease being identified incidentally. Previous studies have indicated a notable decline in the diagnosis of renal cell carcinoma, especially for small asymptomatic masses, during the pandemic. This decrease is likely attributable to the reduction in non-essential imaging, which was then associated with fewer incidental findings [44]. Unsurprisingly, we also found a decrease in incidental discoveries, and a significant increase in the percentage of non-incidental discoveries of KC during the pandemic. Symptomatic presentations of cancer were linked to poorer cancer-specific survival and metastasis-free survival when compared to asymptomatic cases, likely indicating a higher stage of disease at diagnosis [8].

A shift towards the diagnosis of more advanced tumors among different cancers, such as head-and neck cancers, during the pandemic has been reported [10,11,45]; however, there is limited data regarding the distribution of tumor stages in KC between the pre-pandemic and pandemic periods. A study conducted in the UK found no evidence of increased clinical or pathological upstaging in patients who underwent deferred surgery, compared to practices prior to the COVID pandemic [15]. Furthermore, research conducted in the Netherlands and Northern Italy indicated that despite a decrease in new diagnoses during the pandemic, no shift toward more advanced tumors could be detected in the short term [16,46]. The study by Mangone et al. also observed that the overall decline in new cancer diagnoses was primarily attributed to a reduction in T1a and T1b RCC cases, particularly among the elderly [16]. In line with these findings, we did not observe a significant difference in tumor stage distribution, although there was a noticeable trend toward higher cancer stages during the pandemic. Additionally, a greater proportion of patients whose KC was detected incidentally during this time had advanced-stage cancer (stages III–IV). Furthermore, the percentage of patients diagnosed incidentally while undergoing evaluation for chronic conditions—some potentially related to hypertension or diabetes—decreased significantly from 2.5% to 0.7%. Numerous studies indicate that elevated blood pressure can double or triple the risk of renal cell carcinoma, while individuals with diabetes mellitus face a 40% increased likelihood of developing kidney cancer over their lifetime, with prevalence rates of diabetes and hypertension among RCC patients at 20% and 50%, respectively [7,47,48]. Given that elevated BMI, hypertension, and diabetes mellitus are established risk factors for kidney cancer and frequently co-occur [7,47,48], the detection of kidney cancer was likely affected during the pandemic, as the chances of incidental discovery in this vulnerable, high-risk group diminished. Thus, our findings highlight the critical role of incidental detection in high-risk populations and the indirect effects of the COVID-19 pandemic on the timely detection of kidney cancer.

While our study did not focus on treatment modalities, it is worth noting that the surgical management of urologic cancers during the pandemic has raised important questions about access to minimally invasive techniques. As highlighted by Andras et al., the laparoscopic approach may offer a viable alternative in settings where robotic surgery is less accessible or delayed due to resource constraints.

During the pandemic, there was a small but significant decrease in the rate of incidental kidney tumor discovery by urologists. Additionally, our findings revealed that initially consulting a physician other than a urologist notably increased the odds of incidental kidney cancer detection, to a similar extent in both study periods. These results underscore the important role that non-urologist specialists may play in the incidental detection of kidney cancer [49].

The appropriateness and justification of kidney cancer screening have been debated. On one hand, detecting asymptomatic disease can result in stage migration, leading to earlier-stage disease and better treatment outcomes [43,50]. Conversely, due to its comparative rarity, questions about cost-effectiveness and the optimal screening methods raise doubts about the feasibility of introducing widespread KC screening [51,52]. Ultrasound imaging has been proposed as a viable screening method, particularly for high-risk patients such as 60-year-old male patients or those with certain comorbidities, such as hypertension and diabetes [51,53].

Our findings indicated that non-incidental discovery of kidney cancer significantly raised the risk of advanced disease during both study periods, which is unsurprising since asymptomatic, incidental detections are more prevalent in early-stage kidney cancer [8]. However, this relationship was notably weaker during the pandemic, with the odds of having advanced cancer due to non-incidental discovery decreasing from 3.42 to 2.03. This may be explained by a potential increase in the proportion of advanced-stage cancers among incidentally detected cases, possibly reducing the predictive value of symptoms. This phenomenon may underscore the significance of incidental discoveries as a form of “screening” for patients with certain chronic diseases. Supporting this hypothesis, we found that while the presence of symptoms was linked to significantly higher odds of advanced cancer in both study periods, this relationship appeared weaker during the pandemic (OR: 4.51 vs. 2.76), although the difference was not statistically significant. However, the consistent direction of change and alignment with previously published findings suggest a potential trend that warrants further investigation.

The observed decline in incidental kidney cancer detection among patients with comorbidities during the pandemic highlights a critical gap in maintaining routine care for high-risk groups. Disruptions to chronic disease management and diagnostic imaging likely delayed early detection in these patients. To mitigate this, healthcare policies should ensure continuity of routine care and imaging access during crises, especially for those with chronic diseases [54].

## 5. Limitations

This study has several limitations, including its focus on a single center, which may restrict the generalizability of findings to a broader population. Additionally, due to the retrospective design of the study and the reliance on medical records, symptom data were obtained from clinical documentation. This may introduce bias related to subjectivity, variability in documentation practices, or incomplete recording, as the presence and type of symptoms at presentation could be affected by both patient recall and inconsistent reporting in medical records. Non-significant findings should be interpreted with caution. Although consistent directional trends were observed, the study may have been underpowered to detect small but meaningful differences, particularly in subgroup analyses. Additionally, despite adjustment for key covariates, residual confounding from unmeasured factors—such as health-seeking behavior, socioeconomic status, or healthcare access—cannot be excluded.

## 6. Conclusions

Our study highlighted the impact of the COVID-19 pandemic on the demographic and clinical characteristics of kidney cancer patients and revealed unique changes in detection patterns during this period.

We observed a significant increase in the proportion of female patients and those with fewer comorbidities during the pandemic. The overall decline in kidney cancer cases may be attributed to a reduction in incidental detections, where kidney cancer was found unintentionally during imaging for other chronic conditions, such as hypertension and diabetes, as well as a decrease in incidental cases identified by urologists. Additionally, while non-incidental discovery and the presence of symptoms were identified as predictive factors for advanced-stage kidney cancer, their predictive strength diminished notably during the COVID-19 pandemic.

Possible underlying mechanisms for these phenomena include increased health consciousness among women compared to men, heightened fear of COVID-19 infection in patients with multiple comorbidities, and reduced access to healthcare for non-urgent medical conditions. Additionally, a lesser focus on managing common conditions like hypertension and diabetes may have led to fewer incidental detections of kidney cancer, subsequently weakening the relevance of predictive factors for advanced-stage cancer, including non-incidental discovery and the presence of symptoms.

Although our study compares the pre-pandemic and pandemic periods, it sheds light on the proportions and characteristics of incidental versus non-incidental findings, emphasizing the individual significance of these examinations in kidney cancer detection. Health care provided for patients seeking medical attention for unrelated reasons to KC—thus leading to incidental detection—plays a crucial role in detecting a significant proportion of KC cases, as demonstrated by the loss in incidental findings during the pandemic.

These findings may be applicable to other health systems with similar structures, particularly in Central and Eastern Europe, where healthcare access and incidental detection patterns are comparable. In anticipation of future systemic disruptions, we recommend prioritizing the continuity of routine imaging and chronic disease management to preserve incidental cancer detection pathways, especially for high-risk populations.

Our results demonstrate the indirect effect of COVID-19 and underscore the complexities in detecting a cancer type that is often asymptomatic and reveals unexpected impacts on the demographic and clinical characteristics of patients. Further studies are needed to elucidate the underlying reasons for these changes.

## Figures and Tables

**Table 1:** The demographic and clinical characteristics of kidney cancer patients (n = 400) who visited the UP UC during the two study periods: pre-COVID-19 and COVID-19. The Chi-square Test was used to assess statistical significance.

	Before COVID-19		During COVID-19		Chi-Square Test’s *p* Value
	n	*%*	n	*%*	
**Sex**					0.023
**Male** **Female** **Total**	139	*68.1*	112	*57.1*	
	65	*31.9*	84	*42.9*	
	204	*100.0*	196	*100.0*	
**Age**					0.307
**0–49** **50–59** **60–69** **≥70** **Total**	30	*14.7*	27	*13.8*	
	54	*26.5*	57	*29.1*	
	62	*30.4*	71	*36.2*	
	58	*28.4*	41	*20.9*	
	204	*100.0*	196	*100.0*	
**Place of residence**					0.087
**County seat** **City** **Other location** **Total**	54	*26.5*	54	*27.6*	
	60	*29.4*	75	*38.3*	
	90	*44.1*	67	*34.1*	
	204	*100.0*	196	*100.0*	
**Distance from UP UC [km]**					0.048
**≤40** **>40** **Total**	84	*41.2*	100	*51.0*	
	120	*58.8*	96	*49.0*	
	204	*100.0*	196	*100.0*	
**Smoking**					0.555
**No** **Yes** **Total**	145	*71.1*	134	*68.4*	
	59	*28.9*	62	*31.6*	
	204	*100*	196	*100*	
**Charlson Comorbidity Index**					0.048
**≤4** **≥5** **Total**	86	*42.2*	102	*52.0*	
	118	*57.8*	94	*48.0*	
	204	*100*	196	*100*	
**Stage**					0.632
**I** **II** **III** **IV** **Total**	151	*74.0*	135	*68.9*	
	9	*4.4*	12	*6.1*	
	32	*15.7*	38	*19.4*	
	12	*5.9*	11	*5.6*	
	204	*100*	196	*100*	
**Stage**					0.417
**I–II** **II–III** **Total**	160	*78.4*	147	*75.0*	
	44	*21.6*	49	*25.0*	
	204	*100*	196	*100*	
**Type of initial imaging test**					0.088
US * **CT** **MRI** CEUS ** **Plain chest X-ray** **Total**	157	*77.0*	126	*64.4*	
	36	*17.6*	52	*26.5*	
	7	*3.4*	13	*6.6*	
	3	*1.5*	4	*2.0*	
	1	*0.5*	1	*0.5*	
	204	*100*	196	*100*	

* US: Ultrasound; ** CEUS: Contrast-enhanced Ultrasound.

**Table 2:** Factors significantly associated with the incidental discovery of kidney cancer in the pre-pandemic and pandemic periods.

**(A)**						
**STAGE**		**Before COVID-19**		**During COVID-19**		***p***
		**Incidental**		**Incidental**		
		**No**	**Yes**	**No**	**Yes**	**0.001**
**Early** **(I–II)**	n [%]	21 [58.3%]	139 [82.7%]	35 [64.8%]	112 [78.9%]	
**Advanced (III–IV)**	n [%]	15 [41.7%]	29 [17.3%]	19 [35.2%]	30 [21.1%]	
**Total**	n [%]	36 [100.0%]	168 [100.0%]	54 [100.0%]	142 [100.0%]	
**(B)**						
**Initially Consulted Physician**		**Before COVID-19**		**During COVID-19**		***p***
		**Incidental**		**Incidental**		
		**No**	**Yes**	**No**	**Yes**	**0.010**
**Other specialty**	n [%]	19 [52.8%]	125 [74.4%]	29 [53.7%]	106 [74.6%]	
**Urologist**	n [%]	17 [47.2%]	43 [25,6%]	25 [46.3%]	36 [25.4%]	
**Total**	n [%]	36 [100%]	168 [100%]	54 [100%]	142 [100%]	

**Table 3:** Predictive factors of late-stage kidney cancer and incidental discovery in the periods before and during the COVID-19 pandemic.

**(** **A)**				
		**Before COVID-19 OR/CI 95%**	**During COVID-19** **OR/CI 95%**	**Z-** **V** **alue/*p*-** **V** **alue**
**Incidental discovery**	‘Other’ specialist as initially contacted physician	**2.6** [1.240–5.454]	**2.54** [1.318–4.897]	0.046/0.963
**(B** **)**				
		**Before COVID-19 OR/CI 95%**	**During COVID-19** **OR/CI 95%**	**Z-** **V** **alue/*p*-** **V** **alue**
**Advanced-stage**	Non-incidental discovery	**3.42** [1.579–7.424]	**2.03** [1.018–4.035]	0.987/0.324
	Presence of symptoms	**4.51** [1.972–10.321]	**2.76** [1.302–5.829]	0.862/0.386

## Data Availability

The datasets used and/or analyzed during the current study are available from the corresponding author on reasonable request.

## Supplementary Materials

The following supporting information can be downloaded at: https://www.mdpi.com/article/10.3390/cancers17132150/s1, Table S1: Factors with no significant association with incidental discovery of kidney cancer in the pre-pandemic and pandemic periods.

## Abbreviations

The following abbreviations are used in this manuscript:

CCI	Charlson Comorbidity Index
CEUS	Contrast Enhances Ultrasound
EU	European Union
KC	Kidney Cancer
RCC	Renal Cell Carcinoma
STROBE	Strengthening the Reporting of Observational Studies in Epidemiology
UK	United Kingdom
UP UC	University of Pécs Clinical Center Urology Clinic
US	Ultrasound
