# Supplementary material for: Changes in the Characteristics of Kidney Cancer Detection During the COVID-19 Pandemic
Source: Cancers (Basel). 2025 Jun 26;17(13):2150. doi: 10.3390/cancers17132150 (PMC12248619; doi:10.3390/cancers17132150)
Supplement: Supplementary file 1 [file cancers-17-02150-s001.zip › cancers-3669640-supplementary.pdf]

Changes in the Characteristics of Kidney Cancer Detection During the COVID-19 Pandemic

László Rumi, Árpád Szántó, Dániel Bányai, Éva Szabó, Antal Zemplényi, Szabolcs Bellyei, Emese Mátyus, Dóra Hubai, János Girán, István Kiss, Éva Pozsgai and Árpád Boronkai

Table S1: Factors with no significant association with the incidental discovery of kidney cancer in the pre-pandemic and pandemic periods.

	Before COVID-19				During COVID-19				
	Non-incidental		Incidental		Non-incidental		Incidental		
	n	%	n	%	n	%	n	%	p
**Distance from UP UC [km]**									**0.119**
**≤40**	19	*52.8*	65	*38.7*	22	*40.7*	78	*54.9*	
**>40**	17	*47.2*	103	*61.3*	32	*59.3*	64	*45.1*	
**Total**	36	*100*	168	*100*	54	*100*	142	*100*	
Charlson Comorbidity Index									**0.662**
**≤4**	14	*38.9*	72	*42.9*	28	*51.9*	74	*52.1*	
**≥5**	22	*61.1*	96	*57.1*	26	*48.1*	68	*47.9*	
**Total**	36	*100*	168	*100*	54	*100*	142	*100*	
**Age**									**0.648**
**0–49**	4	*11.1*	26	*15.5*	5	*9.3*	22	*15.5*	
**50–59**	12	*33.3*	42	*25.0*	18	*33.3*	39	*27.5*	
**60–69**	9	*25.0*	53	*31.5*	20	*37.0*	51	*35.9*	
**≥70**	11	*30.6*	47	*28.0*	11	*20.4*	30	*21.1*	
**Total**	36	*100*	168	*100*	54	*100*	142	*100*	
**Place of residence**									**0.340**
**County seat**	12	*33.3*	42	*25.0*	14	*25.9*	40	*28.2*	
**City**	12	*33.3*	48	*28.6*	18	*33.3*	57	*40.1*	
**Other loc.**	12	*33.3*	78	*46.4*	22	*40.8*	45	*31.7*	
**Total**	36	*100*	168	*100*	54	*100*	142	*100*	
**Smoking**									**0.520**
**No**	24	*66.7*	121	*72.0*	33	*61.1*	101	*71.1*	
**Yes**	12	*33.3*	47	*28.0*	21	*38.9*	41	*28.9*	
**Total**	36	*100*	168	*100*	54	*100*	142	*100*	
